# Digital Light Processing Bioprinted Human Chondrocyte-Laden Poly (γ-Glutamic Acid)/Hyaluronic Acid Bio-Ink towards Cartilage Tissue Engineering

**DOI:** 10.3390/biomedicines9070714

**Published:** 2021-06-23

**Authors:** Alvin Kai-Xing Lee, Yen-Hong Lin, Chun-Hao Tsai, Wan-Ting Chang, Tsung-Li Lin, Ming-You Shie

**Affiliations:** 1 School of Medicine, China Medical University, Taichung 406040, Taiwan; Leekaixingalvin@gmail.com (A.K.-X.L.); ritsai8615@gmail.com (C.-H.T.); 2 x-Dimension Center for Medical Research and Translation, China Medical University Hospital, Taichung 40447, Taiwan; roger.lin0204@gmail.com (Y.-H.L.); wcewntdy91104@gmail.com (W.-T.C.); 3 The Ph.D. Program for Medical Engineering and Rehabilitation Science, China Medical University, Taichung 406040, Taiwan; 4 Department of Orthopedics, China Medical University Hospital, Taichung 406040, Taiwan; 5 Department of Sports Medicine, College of Health Care, China Medical University, Taichung 406040, Taiwan; 6 Graduate Institute of Biomedical Sciences, China Medical University, Taichung 406040, Taiwan; 7 School of Dentistry, China Medical University, Taichung City 40447, Taiwan; 8 Department of Bioinformatics and Medical Engineering, Asia University, Taichung City 41354, Taiwan

**Keywords:** digital light processing, chondrocyte, poly (γ-glutamic acid), hyaluronic acid, bio-ink

## Abstract

Cartilage injury is the main cause of disability in the United States, and it has been projected that cartilage injury caused by osteoarthritis will affect 30% of the entire United States population by the year 2030. In this study, we modified hyaluronic acid (HA) with γ-poly(glutamic) acid (γ-PGA), both of which are common biomaterials used in cartilage engineering, in an attempt to evaluate them for their potential in promoting cartilage regeneration. As seen from the results, γ-PGA-GMA and HA, with glycidyl methacrylate (GMA) as the photo-crosslinker, could be successfully fabricated while retaining the structural characteristics of γ-PGA and HA. In addition, the storage moduli and loss moduli of the hydrogels were consistent throughout the curing durations. However, it was noted that the modification enhanced the mechanical properties, the swelling equilibrium rate, and cellular proliferation, and significantly improved secretion of cartilage regeneration-related proteins such as glycosaminoglycan (GAG) and type II collagen (Col II). The cartilage tissue proof with Alcian blue further demonstrated that the modification of γ-PGA with HA exhibited suitability for cartilage tissue regeneration and displayed potential for future cartilage tissue engineering applications. This study built on the previous works involving HA and further showed that there are unlimited ways to modify various biomaterials in order to further bring cartilage tissue engineering to the next level.

## 1. Introduction

Cartilage injuries are the main cause of disability in the United States. Cartilage injuries caused by osteoarthritis are projected to affect 30% of the entire United States population by the year 2030 [1]. Cartilage is unlike other connective tissues in that it is mainly avascular. Thus, nutrients and wastes travel via diffusion through the extra-cellular matrix (ECM), which consists of sulfated glycosaminoglycans (such as chondroitin sulfate), glycosaminoglycans (GAGs, such as hyaluronic acid), and type II collagen (Col II) [2]. Due to the avascular nature of cartilage, a single small defect is a major clinical challenge to physicians, since minor lesions often lead to progressive deterioration and impaired regeneration [3]. Current strategies for the treatment of cartilage defects include marrow stimulation (abrasion arthroplasty, microfracture) to allografts or autografts (mosaicplasty) and cell-based therapies. Microfractures used to be the gold standard in the past. They work by introducing controlled lesions into the cartilage to stimulate stem cell differentiation and proliferation [4]. However, clinical results have shown that this type of repair technique is only beneficial in the short- and mid-term because the regenerated fibrocartilaginous tissue exhibits only short-term durability. On the other hand, issues such as limited healthy sources and immune rejection continue to limit allografts and autografts, thus prompting scientists to seek out better alternatives [5].

Cell-based therapies include transplanting chondrocytes directly into the defect or using them in combination with biomaterials as a carrier [6,7]. The development of tissue engineering and 3D printing have made it possible to combine cell-based therapies with biomaterials, which have repeatedly been shown to enhance the formation of hyaline cartilage tissues with improved mechanical, physical, and biological characteristics as compared to previous fibrocartilaginous tissue [8,9,10,11]. In order to achieve efficient tissue regeneration, the biomaterial not only has to serve as a carrier scaffold, but the scaffold must also undergo safe, stable biodegradation in order to avoid toxicity and match the regeneration rate of tissues [12,13,14]. Synthetic polymers have been shown to have better mechanical properties and more stable degradation as compared to natural polymers, but synthetic materials tend to have intermediate degradation products that alter the local pH, and thus cause localized downstream immunological and inflammatory reactions [15,16]. In addition, chondrocytes are an exclusive type of cell only found in cartilage. However, it is extremely difficult to harvest sufficient healthy chondrocytes for transplantation. Furthermore, harvested chondrocytes easily lose their phenotypes if cultured on traditional 2D culture systems [17]. The development of 3D printing has since made it possible to fabricate suitable micro-environments for chondrocytes and stem cells, thus further confirming the use of biomaterials in supporting chondrocytic phenotypes [18].

Hyaluronic acid, otherwise known as hyaluronan (HA), is a linear polysaccharide consisting of repeated D-glucuronic acid and N-acetyl-D-glucosamine and is an important component in the extracellular matrix of cartilage [19]. An important role of HA is maintaining the structural capability of cartilage by retaining water and by interacting with aggrecan and Col II. Patients suffering from osteoarthritis have been noted to have less endogenous HA in their cartilage, thus causing chronic pain due to a lack of lubrication and shock absorbance. HA has gained increasing attention in the area of tissue engineering, especially in the case of cartilage engineering, due to the fact that HA is biocompatible, non-immunogenic, and biodegradable [20]. The HA products come in multiple forms, such as hydrogels, fibers, and meshes. In vitro HA has been reported to increase the synthesis of the ECM, to increase the sensitivity of chondrocytes to growth factors, and to down-regulate matrix metalloproteinases and inflammatory cytokines. However, clinical trial results have revealed that intra-articular HA has a short half-life due to rapid enzymatic breakdown. With a better understanding of the molecular pathway behind the degradation, scientists are now attempting to develop novel HA hydrogels that are able to resist rapid degradation in order to stimulate cartilage repair or regeneration [21].

γ-poly(glutamic) acid (γ-PGA) is a hydrophilic polyanionic polymer that has excellent biodegradability and biocompatibility [22]. γ-PGA is commonly used to modify biomaterials such as polycaprolactone, chondroitin sulfate, and chitosan. In vivo and in vitro results have shown that modification with γ-PGA enhances the mechanical strength of hydrogels and increases the secretion of GAGs from chondrocytes. In addition, chitosan-γ-PGA hydrogels crosslinked using genipin have been reported to have controllable degradation and to exhibit enhanced biological activity as compared to pure chitosan hydrogels [23]. Furthermore, the authors took it a step further to modify the surface of chitosan-γ-PGA hydrogels with elastin, albumin, and poly-L-lysine. The initial results showed that the modified hydrogels had higher porosity and improved mechanical strength and degradation rates, as well as enhanced secretion of Col II and GAGs. 

To the best of our knowledge, there have been no reports in which γ-PGA and HA were combined and assessed for feasibility of use in cartilage tissue engineering. In this study, we demonstrated that the modification γ-PGA-GMA with HA enhanced the physical characteristics and mechanical properties of existing hydrogels. Furthermore, chondrogenic differentiation and proliferation were enhanced, as shown in the increased GAG and Col II secretions and the Alcian blue staining. These results suggest that HA modification is suitable for future cartilage regeneration studies and applications.

## 2. Materials and Methods

### 2.1. γ-PGA-GMA Hydrogel Synthesis

First, 40 g of γ-PGA powder (average molecular weight 1250 kDa, Vedan, Taichung, Taiwan) was added into 400 mL of deionized water and stirred at 50 ℃ to obtain a 10% γ-PGA solution. Next, 19.2 mL of glycidyl methacrylate (GMA, Sigma-Aldrich, St. Louis, MO, USA) was added to the solution and stirred for 6 h at 60 °C for 30 min. The mixture was then centrifuged at 8000 rpm and 40 ℃ for 30 min. Subsequently, the supernatant was collected and placed into a dialysis bag (10K MWCO, Thermo Fisher Scientific, Waltham, MA, USA) for 6 h of dialysis. The γ-PGA-GMA was collected, lyophilized for 24 h, and stored until further usage. Prior to usage, the γ-PGA-GMA was sterilized with 30 min of UV light.

### 2.2. Preparation of Photo-Polymerizable γ-PGA-GMA Solution

Firstly, 0.25% lithium phenyl-2,4,6-trimethylbenzoyl phosphonate (LAP, Compton, UK) was dissolved in phosphate-buffered saline (PBS, Invitrogen, Grand Island, NY, USA) and filtered through a 0.22 μm sieve to remove unwanted products. Then, 10% γ-PGA-GMA with different proportions of HA (0, 1, and 2 wt%), was added to the dissolved LAP to obtain photo-polymerizable γ-PGA-GMA solutions. The solution was then stored in a dark sterile cabinet for further usage.

### 2.3. Characterization of γ-PGA-GMA

The γ-PGA-GMA solution was dissolved in deionized water to obtain a 0.5% γ-PGA-GMA solution and poured into a special glass tube for NMR analysis at a frequency of 500 MHz. In this study, a rheological analyzer (MCR 302, Anton Paar, Graz, Austria) was used to determine the viscosity and curing time of the hydrogel. The predetermined setting was as follows: 25 °C, 0.5 cm above the platform with both ends clamped to a 25 mm plate, and 180 s of 320–500 nm 1.77 W wavelength light source (Omnicure Series 1500, Excelitas Technologies Corporation, Billerica, MA, USA) with a 45% output rate. A supporting mold was fabricated according to ASTM D-638 criteria using the INKREDIBLE bioprinter and 30% Pluronic^®^ F-127 (Sigma-Aldrich, St. Louis, MO, USA). The γ-PGA-GMA hydrogel was then poured into the mold and cured using UV light, and cold PBS was used to dissolve the F-127 mold to obtain the γ-PGA-GMA hydrogel. An EZ-Test (Shimadzu, Kyoto, Japan) desktop testing machine was used to test the tensile strength of the samples. For this test, the prepared hydrogel was first dried under room temperature and weighed to obtain the initial weight. Next, the hydrogels were soaked in deionized water and taken out at different time-points, from 30 min up to 48 h, and dried and re-weighed (Ws). The following formula was used to convert the degree of swelling (Equation (1)):

(1) $$\mathrm{Swelling}\;\mathrm{ratio}:\frac{W_{s}-W_{0}}{W_{0}}\;\times \;100\;\%.$$

### 2.4. Cell Culture

In this study, 3rd to 8th generation human chondrocytes (HCs, ScienCell Research Laboratories, Carlsbad, CA, USA) were used. The HCs were cultured with a commercial medium (#4651, ScienCell Research Laboratories, Carlsbad, CA, USA) containing 500 mL of basal medium, 25 mL of fetal bovine serum, 5 mL of a chondrocyte growth supplement, and 5 mL of a penicillin/streptomycin solution. The HCs were then cultured in a 37 ℃ incubator with 5% CO_2_ with the culture medium changed every two days.

### 2.5. Bioprinting

An 8 × 22 mm supporting framework was fabricated using the INKREDIBLE bioprinter and 30% Pluronic^®^ F-127 (Polyethylene-polypropylene glycol, Sigma-Aldrich, St. Louis, MO, USA). The HCs were then encapsulated into the γ-PGA-GMA hydrogel at a concentration of 1 × 10^6^/mL. The γ-PGA-GMA hydrogel was then poured into a mold and cured using UV light (1.77 W, 12 cm above, and 90 s). Then, cold PBS was used to dissolve the F-127 framework to obtain the γ-PGA-GMA hydrogel.

### 2.6. Cell Proliferation and Viability Assay

After 1, 3, and 7 days of culture, PrestoBlue^®^ reagent (PrestoBlue™ Cell Viability Reagent, Invitrogen, Grand Island, NY, USA) was added into the culture dishes and left to react for 4 h. Then, 100 µL of the solution was pipetted into a fresh 96-well plate for absorbance analysis using a spectrophotometer (Infinite Pro M200, Tecan, Männedorf, Switzerland) at 570 nm wavelength with a reference wavelength of 650 nm. For the viability test, the hydrogels were removed from the culture medium, rinsed with PBS, and treated with live/dead viability/cytotoxicity kits (Invitrogen, Grand Island, NY, USA). A confocal microscope (Leica TCS SP8, Wetzlar, Germany) was used to observe the fluorescent cells. The membranes of the live and dead cells were marked with green and red fluorescence, respectively.

### 2.7. GAG, Collagen I, and Collagen II Quantification

The quantity of GAG, Col I, and Col II secreted from the HCs at different time-points were determined using enzyme-linked immunosorbent assay according to the manufacturer’s instructions. GAG enzyme-linked immunosorbent assay kits (MBS7606393, MyBioSource, San Diego, CA, USA), Col I enzyme-linked immunosorbent assay kits (MBS7607063, MyBioSource, San Diego, CA, USA), and Col II enzyme-linked immunosorbent assay kits (MBS263555, MyBioSource, San Diego, CA, USA) were used to quantify GAG, Col I, and Col II in the HC-laden hydrogels at days 1, 3, and 7. This test was conducted thrice, and the results were averaged.

### 2.8. GAG Staining

After 7 and 14 days of culture, the HC-laden hydrogels were removed and rinsed thrice with PBS. They were then fixed with 4% paraformaldehyde for 1 h, rinsed with PBS, and acidified with H_2_SO_4_ for 30 min. Then, 10 mg/mL of Alcian blue solution was added and left to react for 3 h, after which the HC-laden hydrogels were rinsed with 0.018 M of H_2_SO_4_ and observed using a microscope.

### 2.9. Statistical Analyses

A one-way statistical analysis of variance (ANOVA) was applied to analyze the significance of the between-group differences in each experiment. Determination of the significant deviations of each sample was made using Scheffe’s multiple comparison test. The statistical solutions showed that a *p*-value < 0.05 could be considered statistically significant, as indicated by an *.

## 3. Results and Discussion

### 3.1. Characterization of γ-PGA-GMA

**Figure 1:**
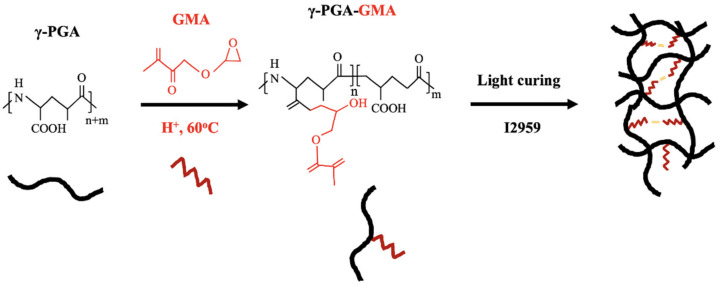
The structural formulas of γ-PGA and a synthesis diagram of γ-PGA-GMA.

γ-PGA-GMA was synthesized via coupling of γ-PGA and glycidyl methacrylate through the Michael addition reaction between these two compounds (Figure 1). This reaction is conducive for use in tissue engineering applications since it is generally a mild and non-toxic reaction. The addition of a photo-polymerizable component made it possible to have control over the mechanical characteristics of the scaffold by simply adjusting the ratio and concentrations of the photo-initiators [24]. Prior to the Michael addition reaction, the γ-PGA was first thiolated with different concentrations of HA so to as allow the subsequent reaction. γ-PGA is a bacterially produced, water-soluble polyamide currently widely used in tissue engineering due to its natural origin and biodegradability [22]. Even so, it is very different from proteins because its peptide bonds consist mainly of α-amino and γ-carboxylic groups. In addition, γ-PGA can be easily modified to better suit clinical needs, such as use as a drug delivery carrier or in biomaterial and thermo-reversible applications.

**Figure 2:**
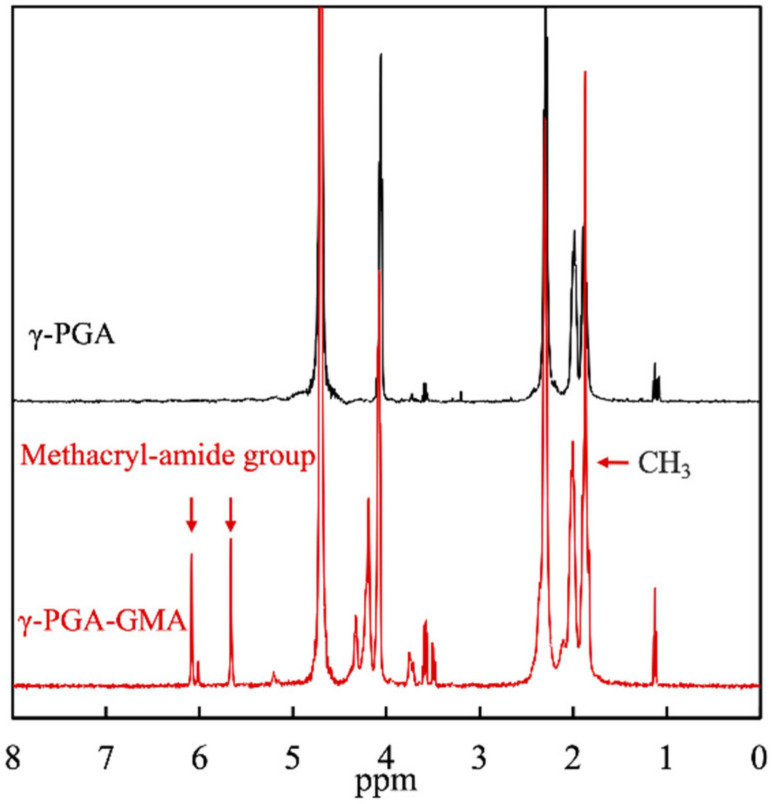
The ^1^H NMR spectra of γ-PGA and γ-PGA-GMA. The peak at 4.1 ppm is the characteristic absorption peak of hydrogen on methine on γ-PGA, whereas 5.5–6.0 ppm is the methylene peak on GMA. The higher the grafting rate of GMA, the larger the peak area.

The chemical structures of γ-PGA and γ-PGA-GMA were characterized using 1H NMR, for which the results are shown in Figure 2. CH3 (1.9 ppm) and various methacryl-amide peaks (5.7 and 6.2 ppm) were present in the γ-PGA-GMA group, which corresponded to the peaks in the glycidyl methacrylate [25]. The presence of these groups indicated the successful conjugation of γ-PGA with glycidyl methacrylate. In addition, the 1H NMR spectrum of the γ-PGA corresponded unambiguously to γ-PGA-GMA, thus further indicating that glycidyl methacrylate was conjugated to the γ-PGA without affecting its original structural characteristics [26]. This was the ideal situation since the goal was to retain the original advantages of γ-PGA and improve the disadvantages of γ-PGA with the addition of glycidyl methacrylate. In this study, the degree of substitution was achieved by comparing the signal strength at 5.7–6.2 ppm (methacryl-amide) to 4.13–4.52 ppm (γ-PGA), and the degree of substitution was at 24.4%. For the subsequent studies, the effects of different concentrations of HA in γ-PGA-GMA on cartilage regeneration were compared and assessed. Therefore, the following results are denoted by HA0, HA1, and HA2, indicating the different concentrations of HA added to the γ-PGA-GMA.

### 3.2. The Mechanical Properties of the γ-PGA-GMA Hydrogel

**Figure 3:**
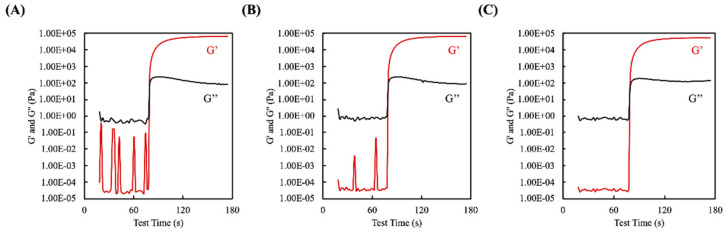
Rheological properties of the three γ-PGA-GMA hydrogel composites: (**A**) H0, (**B**) H1, and (**C**) H2. The cross point between the storage moduli (G′) and the loss moduli (G″) is regarded as the sol–gel transition time-point.

A modular compact rheometer was used to measure the storage and loss moduli of the photo-polymerizable γ-PGA-GMA blended with different concentrations of HA within a 180 s time range. All of the γ-PGA-GMA/HA samples showed consistent storage modulus (G′) and loss modulus (G″) measurements throughout the UV treatment (Figure 3). The results showed that there were no significant differences in curing time at various concentrations of added HA. It can be seen from the figure that the full curing time of the samples was about ~60 s, from the time at which the light was turned on at 20 s to 80 s. There was not much change in the curing time between each test sample, which means that the addition of a small amount of HA did not affect the UV light absorption of the γ-PGA-GMA. This may have been due to the fact that both γ-PGA-GMA and HA have great light transmittance over the specific spectrum of interest. Rheological tests were also carried out at 37 ℃, for which the outcomes were collected at room temperature, which was within the same range as the cell culture temperature, indicating that the available matrix could be applied for the in vitro cell assay [27]. As previously discussed, ideal cartilage substitutes are not only biocompatible but also biomimic the mechanical properties of native cartilage and have adequate strength and tunable swelling that is sufficient to support an efficient tamponade. There has been general study on the mechanical properties of both native cartilage and substituted hydrogels. In the case of the composite hydrogels, the HA concentration had a direct effect on the initial shear storage modulus. Hydrogels formed with the different concentrations of HA were used to determine changes due to the polymeric ratio. In the case of the composite hydrogels, increasing the HA concentration decreased the initial storage modulus. It is possible that this effect could have been due to several factors. In this study, HA alone could not form a hydrogel. Therefore, decreasing the γ-PGA-GMA concentration and increasing the HA concentration led to decreased mechanical properties in these composite hydrogels [28].

**Figure 4:**
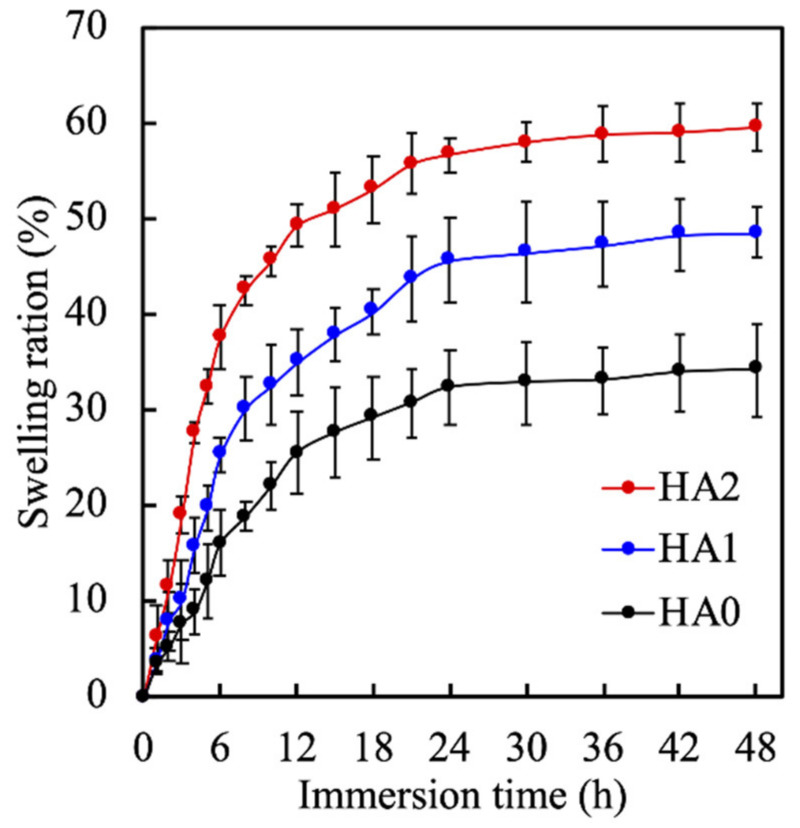
The swelling ratio of γ-PGA-GMA hydrogels with different HA concentrations. Data presented as mean ± SEM, *n* = 6 for each group.

Hydrogel swelling is an important criterion that ensures a good hydrogel for tissue engineering since it can be used to indicate the degree of crosslinking and water content in hydrogels [29]. The swelling ratios of the hydrogels as a percentage of the immersion duration are shown in Figure 4. All the hydrogels reached an equilibrium swelling state at approximately 24 h. However, HA0, 1, and 2 exhibited different equilibrium swelling ratios of 30%, 42%, and 58%, respectively. In addition, all of the hydrogels exhibited rapid water uptake up to the first 12 h of immersion before slowing down and reaching a constant state. This result clearly indicated that the swelling properties of the γ-PGA-GMA hydrogels depended strongly on the crosslinking conditions. The swelling ratios are related to the degree of crosslinking, where a reduction in the crosslinking density resulted in a less restrained network, allowing the hydrogels to swell until equilibrium was established [30]. This result was similar to another study that demonstrated that increases in the content of the highly hydrophilic polymer HA increased water retention [31]. In addition, these results indicated that an HA component may increase the swelling behavior of γ-PGA-GMA hydrogel better mainly due to the high water absorbency of HA [32]. Reports have previously stated that increasing the polymer concentration significantly enhances the equilibrium swelling ratio of hydrogels, which is similar to the results discussed above for the present study.

**Figure 5:**
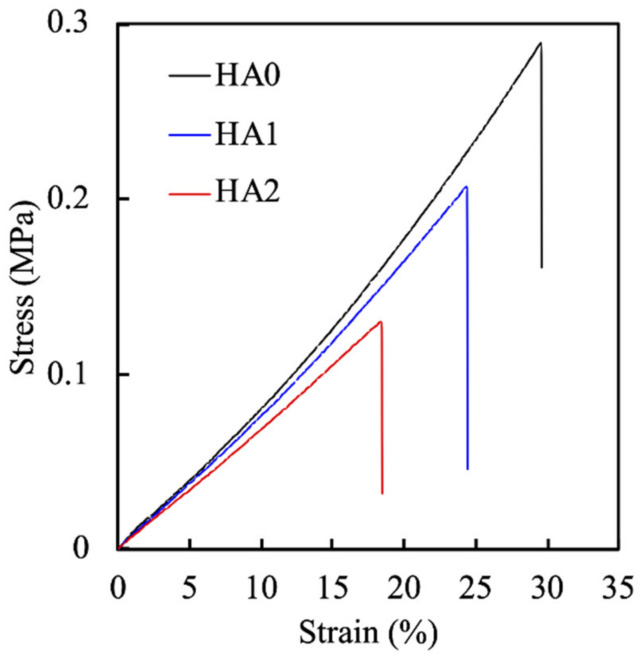
The stress-strain tensile curves of γ-PGA-GMA hydrogels with HA.

The ratio of the polymers not only influences the equilibrium swelling ratio but is also known to affect mechanical properties of γ-PGA-GMA hydrogels. The mechanical properties of the hydrogels were thus evaluated and presented in terms of a stress-strain curve, as shown in Figure 5. As shown, increasing the concentrations of HA negatively influenced the strain, with HA0, 1, and 2 increasing the strain by 30%, 25%, and 18%, respectively. In addition, the tensile strength of the γ-PGA-GMA hydrogels decreased from 288 to 130 kPa with increases in the HA content, indicating that the decrease in HA content enhanced the stiffness of γ-PGA-GMA hydrogels. The γ-PGA-GMA solid content is not only an important factor related to the swelling property but is also a critical factor for the mechanical properties of γ-PGA-GMA hydrogels [33]. In addition, the tensile modulus was also noted to decrease with increases in the HA concentrations. This is an interesting phenomenon that suggests that the polymer concentration is more important than the crosslinking conditions. A report made by Yang et al. stated that γ-PGA is weaker when the concentration is higher because the stronger interactions between the γ-PGA hydrogen bonds lead to weaker dissipating energy, thus making it weaker [34].

### 3.3. Cell Proliferation in HC-laden γ-PGA-GMA Hydrogels

**Figure 6:**
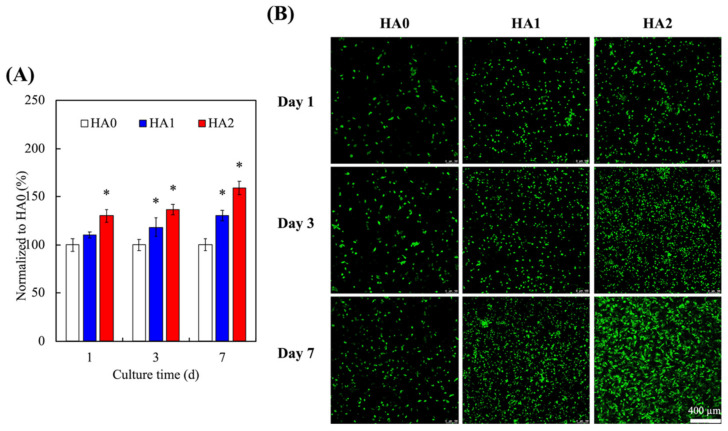
(**A**) Quantification and (**B**) the live/dead assay of HC-laden γ-PGA-GMA hydrogels with various HA concentrations for different days. * indicates significant difference (*p* < 0.05) from HA0. Data are presented as mean ± SEM, *n* = 6 for each group. The scale bar is 400 µm.

A good hydrogel should be able to support cellular proliferation and differentiation [35,36,37]. γ-PGA is a naturally occurring polypeptide with unique similarities to the ECM of native tissues [38]. In recent years, HCs have gained popularity in the field of tissue engineering due to their ability to differentiate into desirable phenotypes. The cellular viability of the encapsulated HCs in the γ-PGA-GMA hydrogels in the present study were evaluated and quantified using live/dead assays, for which the results are shown in Figure 6, where it can be seen in Figure 6A that HA2 had a significantly higher amount of cellular proliferation at all time-points as compared to HA0. On the other hand, HA1 had slower rates of proliferation, with only significantly higher amount of cellular proliferation after 3 days of culture as compared to HA0. In addition, the results for the live/dead staining shown in Figure 6B, where the live cells were stained green and the dead cells were stained red, show that HA2 had more live cells at all time-points as compared to HA0. There were also little or no dead cells in any of the groups. These HA-containing γ-PGA-GMA hydrogels were demonstrated to support HC proliferation and differentiation behavior. In previous studies, γ-PGA-GMA hydrogels have been proven to regulate the adherence and growth of cell lines and to support the maintenance of several primary cell activities [39]. Moreover, the γ-PGA-GMA hydrogels contained a 3D macromolecular network, supporting a high-water-content and 3D microenvironment [40]. In addition, HA hydrogel studies are not only for articular cartilage repair applications, but also indicate that the system needs growth factors for cell growth [41]. The results of previous studies showed that HA-containing γ-PGA-GMA hydrogels have excellent cyto-compatibility and have potential for further research.

### 3.4. In Vitro Cartilage Tissue Formation in γ-PGA-GMA Hydrogels

**Figure 7:**
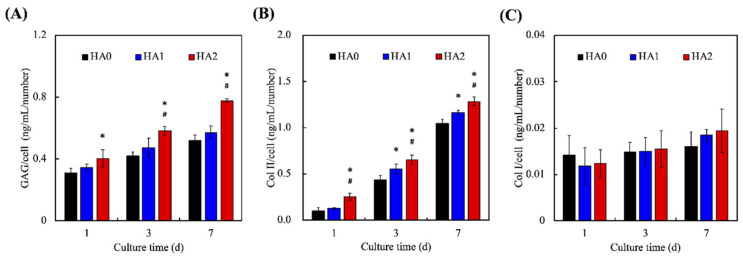
The chondrogenic-related protein of (**A**) GAG, (**B**) Col II, and (**C**) Col I of HC-laden γ-PGA-GMA hydrogels with different HA concentrations for different time-points. * indicates a significant difference (*p* < 0.05) from the H0 group. # indicates a significant difference (*p* < 0.05) from the H5 group. Data are presented as mean ± SEM, *n* = 6 for each group.

The levels of secreted GAG and Col II were evaluated, as shown in Figure 7, where it can be seen that HA2 had a significantly increased amount of GAG on days 1, 3, and 7 as compared to HA0. In addition, HA2 was noted to have a significantly increased amount of GAG on days 3 and 7 as compared to HA1. HA2 had 35 ng/mL, 60 ng/mL, and 82 ng/mL of GAG, while HA0 had 30 ng/mL, 40 ng/mL, and 55 ng/mL on days 1, 3, and 7, respectively. On the other hand, HA2 was noted to have a significantly increased amount of Col II on days 1, 3, and 7 as compared to both HA0 and HA1. HA2 had 30 ng/mL, 75 ng/mL, and 150 ng/mL of Col II on days 1, 3, and 7, respectively, as compared to HA0, which had 10 ng/mL, 45 ng/mL, and 100 ng/mL. GAG comprises a group of negatively charged carbohydrates that make up cell surfaces and the ECM [42]. GAG is involved in the binding of growth factors and cytokines, which then regulate and enhance cellular activities, such as migration, differentiation, and proliferation [43]. In addition, GAG has an important role to play in maintaining the functions and structure of cartilage. GAG has been shown to be rearranged into proteoglycans in cartilage by having core proteins bonded onto its surface, thus making it highly anionic. This characteristic allows cartilage to be resistant to compression through binding and organizing water molecules [40]. The protein expression data indicated that Col II is highly up-improved after 7 days in the γ-PGA-GMA hydrogels, and Col I is almost obliterated. On the other hand, Col II has been widely recognized as one of the critical and most indispensable collagenous compounds in native cartilage. Furthermore, Col II has been shown to play an important role in influencing the development and maturation of chondrocytes [44]. Recently, researchers have attempted to use Col II as a scaffold or to even coat Col II onto existing scaffolds. Their results indicate that the presence of Col II alone is able to enhance the secretion of the cartilage matrix from chondrocytes [21]. A recent study by Dai et al. showed that injection of squid Col II into osteoarthritic joints enhanced cartilage regeneration by activating M2 macrophages and inhibiting apoptosis and hypertrophy in chondrocytes [45]. Both GAG and Col II play important roles in and are critical components of healthy cartilage. In addition, they have overlapping roles in terms of influencing cellular activities such as the differentiation and migration of chondrocytes.

**Figure 8:**
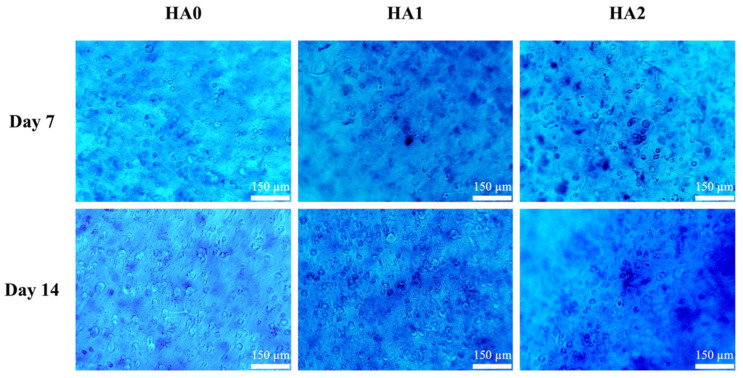
Photographs of Alcian blue staining representing sulfated glycosaminoglycans (chondroitin sulfate) displayed in the matrix of the HCs/HA-containing γ-PGA-GMA hydrogel construct cultured in vitro on days 7 and 14. The scale bar is 150 μm.

Alcian blue staining after 7 and 14 days of culture was carried out to assess the capability of the hydrogels to promote cartilage regeneration, for which the results are shown in Figure 8. There was progressive intensity in the staining for all groups from days 7 to 14, thus indicating that the chondrocyte cells were secreting GAG and glycoproteins, as evidenced by the positive Alcian blue staining. However, as compared to HA0, the chondrocytes cells in HA2 had more intense staining surrounding the cells, thus indicating that the cells were secreting more GAG in the HA2 groups. The role of HA in cartilage development has been well studied and understood. In the field of tissue engineering, Burdick et al. showed that HA hydrogels provide stem cells and chondrocytes with a favorable micro-environment for cartilage differentiation and cellular proliferation (PMID: 19193129). Further studies indicated that presentation of HA to stem cells at different stages of regeneration allowed for different responses. A separate study conducted by Lee et al. showed that injected mesenchymal stem cells and HA regenerated more cartilage-like tissues after 28 weeks of implantation (seen from the positive Masson trichrome, toluidine blue, and Col II staining) as compared to mesenchymal stem cells alone or with normal saline [41]. Together with our study, these results show that HA is an essential tool in future tissue engineering. However, the exact mechanism still waits for further clarification.

In conclusion, we showed that the modification of γ-PGA-GMA hydrogels with HA enhances both the physical characteristics and mechanical properties of existing hydrogels. Furthermore, chondrogenic differentiation and proliferation were enhanced, as evidenced from the enhanced GAG and Col II secretions and Alcian blue staining. These results suggest that HA modifications are suitable for future cartilage regeneration studies and applications. Therefore, we considered HA-containing γ-PGA-GMA hydrogels embedded with HCs that were from the ECM in an ex vivo cartilage regeneration model, since it was possible to estimate the properties of HA-containing γ-PGA-GMA hydrogels in an environment that biomimics natural cartilage.

## 4. Conclusions

In summary, this study demonstrated successful fabrication of different concentrations of hyaluronic-acid-modified γ-PGA-GMA hydrogels. The XRD and FTIR results demonstrated that the modifications were successful and that the modifications were able to retain the initial structural characteristics of both biomaterials. Storage modulus, loss modulus, and curing time were not affected by the concentrations of HA. However, increasing concentrations of HA were found to cause a decline in mechanical properties such as the tensile modulus, but this led to improvements in attaining swelling equilibrium in the hydrogels. Further studies showed that the HA2 groups exhibited significantly enhanced cellular proliferation and secretion of cartilage-regeneration-related proteins such as glycosaminoglycan (GAG) and collagen II (Col II) at all time points when compared to HA1 and HA0. Furthermore, HA2 had higher intensities of Alcian blue staining as compared to the rest of the groups, thus strongly suggesting that modification of γ-PGA-GMA with HA may be a feasible option in future cartilage regeneration studies. In this study, we showed that it was possible to mimic native micro-environments for human cartilage cells by modifying γ-PGA-GMA with HA and that this could be used as a platform for subsequent cartilage regeneration studies.

## Data Availability

Data are available in a publicly accessible repository.
